# One more trip to Barcetona: on the special status of visual similarity effects in city names

**DOI:** 10.1007/s00426-023-01839-3

**Published:** 2023-06-23

**Authors:** Manuel Perea, Melanie Labusch, María Fernández-López, Ana Marcet, Eva Gutierrez-Sigut, Pablo Gómez

**Affiliations:** 1 Universitat de València, Av. Blasco Ibáñez, 21, 46010 Valencia, Spain; 2 Centro de Investigación Nebrija en Cognición, Universidad Nebrija, Madrid, Spain; 3 University of Essex, Essex, UK; 4 California State University, San Bernardino, Palm Desert Campus, San Bernardino, USA

## Abstract

Previous research has shown that, unlike misspelled common words, misspelled brand names are sensitive to visual letter similarity effects (e.g., amazom is often recognized as a legitimate brand name, but not amazot). This pattern poses problems for those models that assume that word identification is exclusively based on abstract codes. Here, we investigated the role of visual letter similarity using another type of word often presented in a more homogenous format than common words: city names. We found a visual letter similarity effect for misspelled city names (e.g., Barcetona was often recognized as a word, but not Barcesona) for relatively short durations of the stimuli (200 ms; Experiment 2), but not when the stimuli were presented until response (Experiment 1). Notably, misspelled common words did not show a visual letter similarity effect for brief 200- and 150-ms durations (e.g., votume was not as often recognized as a word than vosume; Experiments 3–4). These findings provide further evidence that the consistency in the format of presentations may shape the representation of words in the mental lexicon, which may be more salient in scenarios where processing resources are limited (e.g., brief exposure presentations).

## Introduction

Despite the enormous number of words stored in the readers' lexicon and the great variability of their visual forms (e.g., house, House, **HOUSE**, house, etc.), we can accurately recognize a written word in only a few hundred milliseconds. To explain this remarkable ability, most current models of visual-word recognition and reading in alphabetic languages assume that perceptual factors are quickly distilled into abstract representations (i.e., font, size, CASE and color become incidental features; see Grainger, 2018; Grainger & Dufau, 2012). This process of abstraction has been implemented in hierarchically-based neural models of visual word recognition, such as those by Dehaene et al. (2005) and Grainger et al. (2008), in which a layer of abstract letter detectors is the driving force behind lexical access (see Agrawal et al., 2020; Hannagan et al., 2021, for alternative accounts).

A consequence of this assumption is that visual letter similarity should only have a minimal impact on lexical access. The logic is that its effect should be limited to the initial stages of letter encoding, dissipating quickly in the process of visual word recognition (see Carreiras et al., 2013; Gutierrez-Sigut et al., 2019 for electrophysiological evidence using masked primes). Indeed, simulations on a leading computational model of word recognition such as the spatial coding model (Davis, 1999), using the default parameters, show that lexical decisions to the pseudoword CAMEPA, in which the letter R from its base word (CAMERA) was replaced with the visually similar letter P, produces the same lexical decision times (in processing cycles) as the pseudoword CAMESA, in which R was replaced with the visually dissimilar letter S (i.e., 118 cycles in both cases). Empirical evidence with misspelled common words corroborates this view. For instance, in lexical decision experiments, response times and error rates are similar for pseudowords like viotin (where the letter “l” from violin was replaced with the visually similar letter “t”) and viocin (e.g., Perea & Panadero, 2014, Perea et al., 2022; see Gutierrez-Sigut et al., 2022, for electrophysiological evidence). If the visual appearance of letters played a role in how quickly and accurately people recognize words, we would have expected to observe a visual similarity effect. This effect would have resulted in more errors or slower “no” responses for visually similar pseudowords, such as viotin compared to visually dissimilar pseudowords, like viocin. Indeed, the lack of differences between the response times to pseudowords like viotin versus viocin has often been considered a key marker of abstract, orthographic processing (see Ziegler et al., 2013). 

However, at least one type of written word has consistently shown strong visual similarity effects for its misspellings: logotypes (i.e., a logo displaying a company name or initials). In a task in which participants had to indicate whether the presented logotype was genuine or not, Pathak et al. (2019) found substantially longer response times and more errors to visually similar misspelled logotypes (e.g., tacebook, where “f” from facebook was replaced with the visually similar letter “t”) than to visually dissimilar misspelled logotypes like xacebook (see Perea et al., 2022, for a replication with a different set of logotypes). This pattern was explained by a key feature of logotypes: their homogeneity in format. Unlike common words, which can vastly vary in appearance, logotypes are usually presented in a consistent font, style, and layout across different applications. After all, logotypes are designed to be easily recognizable and rely on visual information to achieve this recognition (Foroudi et al., 2017). Notably, visual letter similarity effects are also strong when presenting the misspelled logotypes in plain format (e.g., using Times New Roman; Perea et al., 2022), thus suggesting that the lexical representations of brand names may retain some perceptual elements (see Perea et al., 2021, for a comparison of transposed-letter effects in brand names vs. logotypes). Indeed, brand names are identified faster when presented in their typical letter case (e.g., IKEA faster than ikea; amazon faster than AMAZON; see Gontijo & Zhang, 2007; Perea et al., 2015). In contrast, this pattern does not occur for common words: PHARMACY, although often capitalized, is no more readily identified than pharmacy (Perea et al., 2018).

The robust effect of visual letter similarity for misspelled brand names suggests that some lexical representations may retain some sensitivity to visual information throughout the processing stream. Thus, one might argue that when the same visual form of the representations is often repeated across the various encounters—as occurs with logotypes and brand names, the generalizations created by abstracting over episodes in the “prototypical” lexical representation may contain some visual information (i.e., a weakly episodic account; see Tenpenny, 1995).

In this study, we investigated whether another category of words with a relatively high degree of homogeneity of presentation—city names—also shows a special sensitivity to visual letter similarity effects. City names are often presented in a standardized format, with the first letter of each word capitalized and the rest in lowercase (e.g., Barcelona). Furthermore, city names are encountered more frequently in printed materials, such as maps, travel guides, news, forums, or road signs, which tend to use a restricted set of fonts, colors, or layouts. For instance, the city names used in the present Experiments 1–2 had a considerably higher average frequency in the book/web database than in the subtitle database—where the latter is thought to reflect “everyday language” (M = 28.5 vs. 3.9 per million, respectively in the Duchon et al., 2013, EsPal databases in Spanish). In contrast, for the common words in Experiments 3–4, the frequency of appearance in books vs. informal contexts was comparable (book/web database: M = 54.7 and 57.01 per million in Experiments 3 and 4, respectively; subtitle database: M = 60.6 and 44.7 per million in Experiments 3 and 4, respectively; Duchon et al., 2013).

While the books and other printed (or online) materials in which city names are encountered may feature varying fonts and letter case, they are still more homogeneous that for common words, which may be presented in more informal contexts and in handwritten text. Moreover, visual information in city names is likely to be more homogeneous, take for example the advice to writers to use clear and legible penmanship when handwriting city names in postal addresses. Thus, the homogeneity in the format of city names may make them particularly sensitive to visual information during word identification.^1^ In contrast, due to the occurrence of common words in various written texts and contexts, their specific perceptual features may not be easily represented in their lexical entries (i.e., their lexical representations would be a result of the generalization across many different visual forms; see Tenpenny, 1995). As a result, visual similarity is unlikely to affect the recognition of misspelled common words.

The main aim of the present experiments was to test whether visual information plays a role in the identification of pseudowords that resemble city names. For Experiments 1 and 2, we created two misspelled versions of each city name by replacing a middle letter with either a visually similar letter (e.g., visually similar pseudoword, Barcetona from Barcelona) or a visually dissimilar letter (e.g., visually dissimilar pseudoword, Barcesona). We employed Simpson et al. (2013) letter visual similarity matrix to establish the degree of visual similarity of the misspelled letter with the original letter. The participants' task was to decide whether a given sequence of letters formed a correctly spelled word or not—they were also told that the set of words was composed of city names. The visual similarity effect was operationalized as the difference in response times and accuracy between visually similar pseudowords and visually dissimilar pseudowords. Experiment 1 used a standard setup in which each stimulus was displayed until the participant responded. In Experiment 2, we shortened the exposure duration to 200 ms to induce participants to make “word” decisions without a perfect match between the stimulus and the lexical entries. The logic of Experiment 2 was to maximize the chances of finding visual letter similarity effects for misspelled words (if any) in the absence of a careful post-access spelling check—note that visual letter similarity effects can occur in the first moments of processing (e.g., obiect-OBJECT producing faster responses than obaect-OBJECT in masked priming: Marcet & Perea, 2017, 2018; see also Lally & Rastle, 2022, for evidence with the Reicher–Wheeler task). Experiments 3 and 4 examined whether a limited viewing time could elicit a visual letter similarity effect with misspelled common words (e.g., votumen vs. vosumen; base word: volumen [volume]).

The predictions of Experiment 1 were the following: If the mental representation of city names retains some visual information, we would expect more errors or longer response times for a visually similar pseudoword like Barcetona than for a visually dissimilar pseudoword like Barcesona (i.e., a visual similarity effect). This result would support the idea that visual information may be preserved in the word recognition system for certain types of stimuli, thus posing problems for accounts of visual-word recognition that assume that lexical access is derived only from abstract representations. Alternatively, if there is nothing special about city name identification, we expect similar response times and accuracy for pseudowords like Barcetona and Barcesona. In this latter scenario, we would expect a null effect of visual letter similarity, as occurs with misspelled common words and as predicted by the leading models of visual-word recognition.

## Experiment 1 (misspelled city names, standard setup)

The experiment was pre-registered in the OSF (https://osf.io/js5r7/?view_only=68bea8601f394402b11d08b1b42ab919). The stimuli, data, scripts, and outputs of this and the following experiments are available at: https://osf.io/drsvu/?view_only=c3bd69188768472f86fc6b721997950f

### Method

#### Participants

We recruited 78 native speakers of Spanish (mean age = 29.7 years, SD = 10.1), of which 48 self-identified as female. This sample size guaranteed more than 2,000 observations per condition, following the guidelines of Brysbaert and Stevens (2018). None of the participants had reading difficulties and all had a normal or corrected to normal vision. Participants received monetary compensation through the online-recruitment platform Prolific Academia (https://www.prolific.co) and gave informed consent for their participation. The Ethics Committee for Experimental Research of the Universitat de València approved this series of experiments.

#### Materials

We selected 52 well-known city names from Spain and worldwide to act as base words. To make sure that the city names were familiar to the participants, a group of ten university studies who did not participate in the experiment scored an initial pre-selected list in a 1–5 familiarity Likert scale—the selected city names had average familiarity values above 4.3. These city names were between 5 and 9 letters long. Their mean word frequency was 4.06 (range 0.01–6.12) in the Spanish subtitle database (Duchon et al., 2013). As indicated earlier, the book/web database may offer a better indication of their word frequency (M = 29.26 per million, range 1.08–208.4) and their mean orthographic neighborhood (operationalized as the mean Orthographic Levenshtein Distance of the 20 nearest neighbors; OLD20) was 2.39 (range 1.35–3.95) in the Spanish database EsPal (Duchon et al., 2013). For each city name, we created two pseudowords, one in which we replaced a middle consonant letter with a visually similar consonant letter (e.g., *Barcetona* from Barcelona) and one in which we replaced the same internal consonant with a visually dissimilar consonant letter (e.g., *Barcesona*). The average similarity of the original letter with the visually similar and visually dissimilar condition was 4.13 (range 2.77–5.33) and 1.31 (range 1.07–1.83), respectively, in the Simpson et al.'s (2013) visual letter similarity matrix. The average mean log bigram frequency in Spanish was similar for the two sets of misspelled items (2.11 vs. 2.06, respectively, *p* = 0.26; Davis & Perea, 2005). All pseudowords were pronounceable and orthographically legal. The base word was the only neighboring city name for the pseudowords. For the task, we also selected another set of 52 city names—of similar length as the initial dataset—that was presented correctly written. Their mean word-frequency in the Spanish book/web database (Duchon et al., 2013) was 27.86 (range 1.34–516.5) and their mean OLD20 was 2.40 (range 1.00–4.00). To ensure counterbalancing of the two types of pseudowords across participants, we created two stimulus lists using a Latin-square design. For instance, *Barcetona* would be assigned to List 1 and *Barcesona* to List 2. In each list, participants received 26 visually similar misspelled city names and 26 visually dissimilar misspelled city names—note that they were presented with 52 correctly written city names.

#### Procedure

The experiment was designed with Psychopy 3 (Peirce et al., 2022; for benchmarks on performance, see Bridges et al., 2020) and hosted by its corresponding online server Pavlovia (https://www.pavlovia.org). As the experiment took place online, all participants were instructed to remain in a quiet place without distractions for the duration of the experiment. In the beginning, participants filled out a demographical questionnaire through the platform LimeSurvey (https://www.limesurvey.org), before being redirected to the experiment. The experimental session commenced with 12 practice trials, during which participants were provided with feedback regarding the accuracy of their responses. In the experimental phase, each participant went through 104 trials, and no feedback was provided. At the beginning of each trial, a fixation cross was presented for 500 ms. This was followed by the stimulus until the participant’s response (or until a deadline of 2000 ms). Participants were instructed to determine whether the presented item was a correctly spelled word or not, as swiftly and accurately as possible. To facilitate response times, they were advised to place their right and left index fingers on the "M" (yes) and "Z" (no) keys on their keyboard. Participants were also told that the word stimuli consisted of city names and were provided with a set of exemplars for clarification in the instructions. We recorded both the latency and the response key for each trial. The experiment lasted less than 10 min.

### Results and discussion

Very fast responses (less than 250 ms [0 trials]) and error responses were removed from the response time analyses. It should be noted that if participants did not respond within the 2-s deadline, this was considered an error. While the discussion in this paper is focused on the differences in RT, the interested reader can see the overall performance in the mean response times and accuracy for visually similar and dissimilar misspelled city names in the tables (Table 1 for Experiment 1).

**Table 1:** Mean correct response times (in ms) and error rates (in percentage) for the city names and the misspelled city names in Experiment 1 (exposure duration until response)

City name	Visually similar misspelled city name	Visually dissimilar misspelled city name	Visual similarity effect
770 (9.9)	794 (9.8)	793 (3.8)	1 (6.0)

We created separate Bayesian linear mixed-effects models, using the brms package (Bürkner, 2017) in R (R Core Team, 2022) to analyze accuracy (fitted with a Bernoulli distribution) and response times (fitted with an exGaussian distribution). The only fixed factor in the models was Visual similarity (similar [− 0.5], dissimilar [0.5]), and the resulting maximal random structure model was:

a $$ {\text{Dependent}}\;{\text{Variable }}\; \sim \;{\text{Visual}}\;{\text{Similarity}}\;{\text{ + }}\;\left( {1\; + \;{\text{Visual}}\;{\text{Similarity}}\left| {{\text{Subject}}} \right.} \right)\; + \;\left( {1\; + \;{\text{Visual}}\;{\text{Similarity}}\left| {{\text{Item}}} \right.} \right) $$

**Fig. 1:**
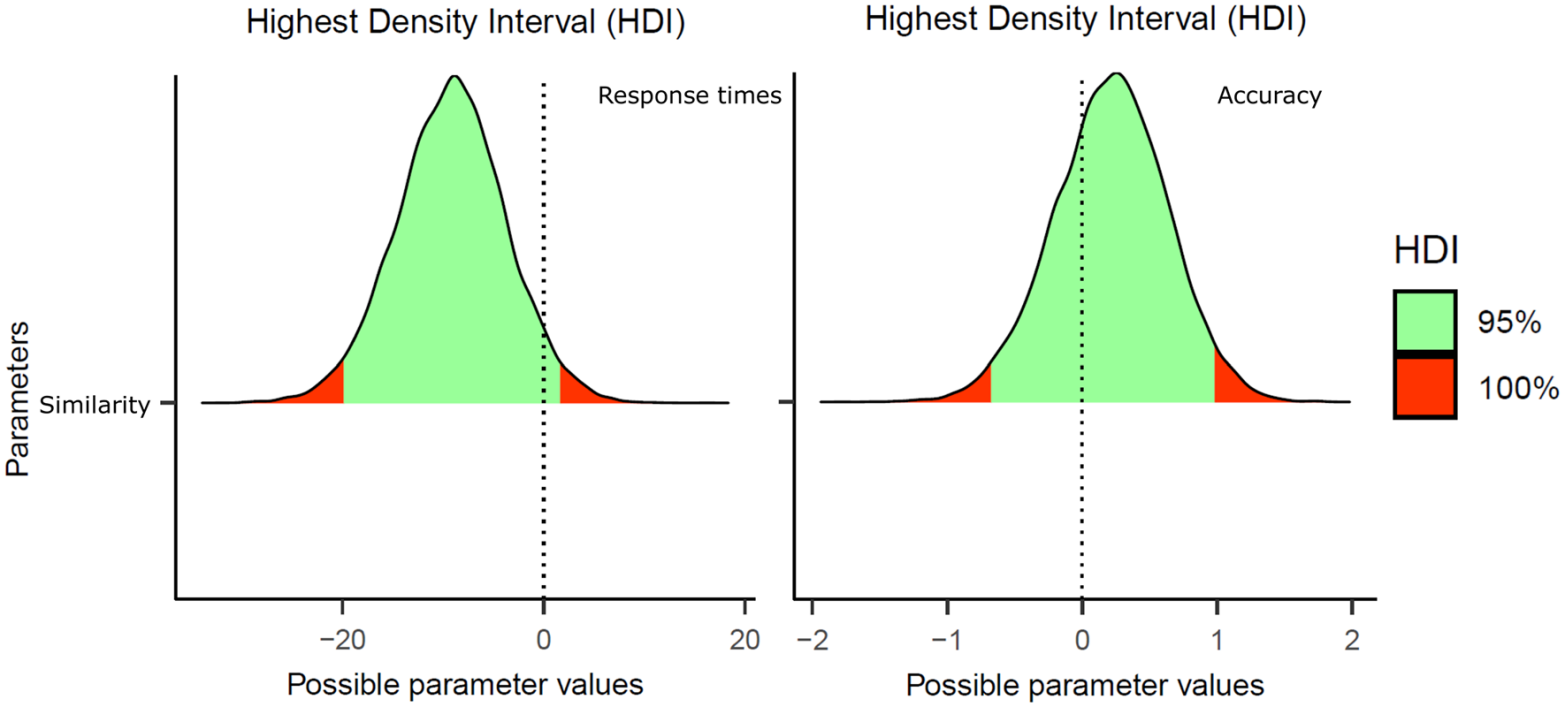
Posterior distributions of the visual letter similarity effect in Experiment 1 (Response Times in the left panel, Accuracy on the right panel). The green areas correspond to the 95% credible intervals

We ran 5000 iterations across four chains—the first 1000 were considered warm-ups. The four chains converged adequately (all *R̂*s = 1.00). The Bayesian linear-mixed effects models' output includes an estimate for the fixed effect (*b*; this would be the median of the posterior distribution of the estimate), its standard error, and its 95% credible interval (CrI). Following the pre-registration protocol, we interpreted that there was a visual similarity effect when the credible interval of its estimate did not cross zero. Figure 1 shows the posterior estimates and the 95% credible intervals for both RTs and accuracy data.

#### Response times

**Fig. 2:**
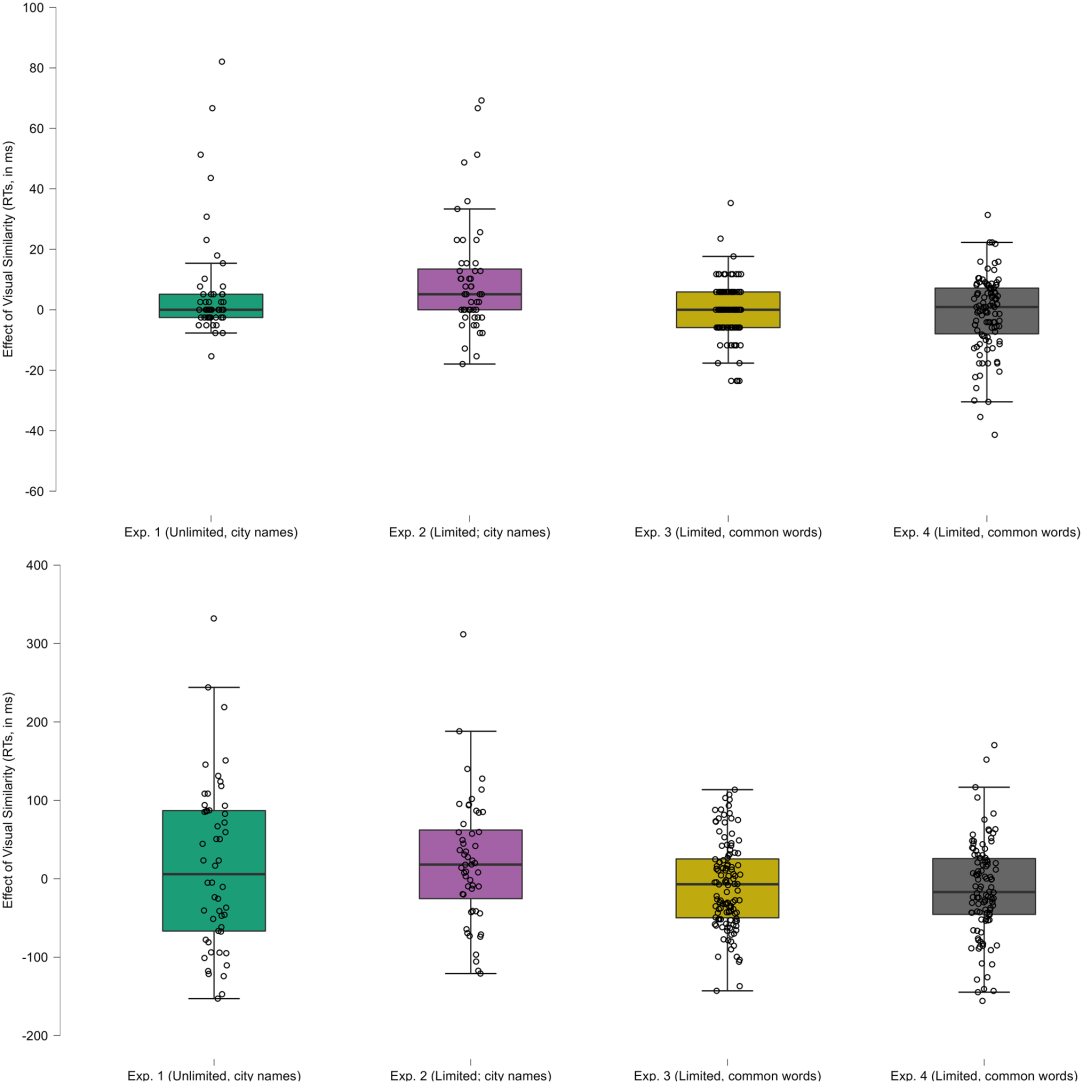
Visual letter similarity effect, averaged by items, in the accuracy data (upper panel) and the latency data (lower panel) for Experiments 1–4

The latency analyses did not reveal an effect of visual letter similarity for misspelled city names, *b* = − 9.20, *SE* = 5.44. 95% CrI [− 20.06, 1.51]. The small difference in the estimates was because, while the by-subjects difference was 1 ms, the by-item effect was 16.5—this was due to a few items that generated a large visual letter similarity effect (see the middle Panel of Fig. 2).

#### Accuracy

The accuracy analyses showed numerically smaller accuracy rates (around 6%) for the visually similar than for visually dissimilar city names. However, not only the 95% credible interval crossed zero, *b* = 0.21, *SE* = 0.42, 95% CrI [− 0.64, 1.02], but the parameter estimate fell within the 50% credible interval (see the middle panel of Fig. 2). This pattern suggests that the apparent numerical difference could have just been due to a small subset of items. We conducted an exploratory by-item analysis via a box plot to examine this. As hypothesized, most of the misspelled items did not show an effect (see the left panel of Fig. 2; e.g., the median was 0).

The present experiment showed that misspelled city names did not exhibit a reliable effect of visual letter similarity. This pattern is in line with the findings with misspelled common words (e.g., RT [accuracy] to votume ≈ RT [accuracy] to vosume; see Gutierrez-Sigut et al., 2022; Perea & Panadero, 2014; Perea et al., 2022). These results are consistent with the idea that lexical access for city names, like common words, is primarily driven by abstract representations (e.g., Dehaene et al., 2005; Grainger et al., 2008).

Nonetheless, there is a possibility that the lexical representations of city names could maintain some sensitivity to sensory information but that this sensitivity was obscured by a post-access spelling check. One option to minimize the impact of this presumed verification stage would be to present the stimuli relatively briefly. In this scenario, participants would make “word’ responses based on a more lenient criterion between the visual input and the stored representations of the city names, maximizing the chances of finding a visual letter similarity effect, if any (see Grossberg & Stone, 1986; Paap et al., 2000; Perea et al., 2005). To examine this hypothesis, Experiment 2 used the same stimuli as Experiment 1, but they were presented for only 200 ms and subsequently masked with hash marks.

If brief exposure durations unveil the visual component behind the representations of city names in the mental lexicon, we would expect worse performance for pseudowords like Barcetona than Barcesona (i.e., an effect of visual similarity). Alternatively, if city names are represented similarly as common words (i.e., as abstract representations), we would expect a null effect of visual letter similarity for misspelled city names (i.e., as in Experiment 1).

## Experiment 2 (misspelled city names, brief presentation)

The experiment was pre-registered in the OSF (https://osf.io/xbczm/?view_only=3125ab7097ee48aab4a5115fa6971822).

### Method

We recruited a new sample of 78 participants from the same population as in Experiment 1 (mean age = 31.1 years, SD = 10.3, 33 self-identified as women). The materials were the same as in Experiment 1. The procedure was the same as in Experiment 1, except that the items were presented for 200 ms and masked with a series of hash marks. The instructions were parallel to Experiment 1, except that participants were told that the items would be presented briefly and followed by a pattern mask.

### Results and discussion

**Fig. 3:**
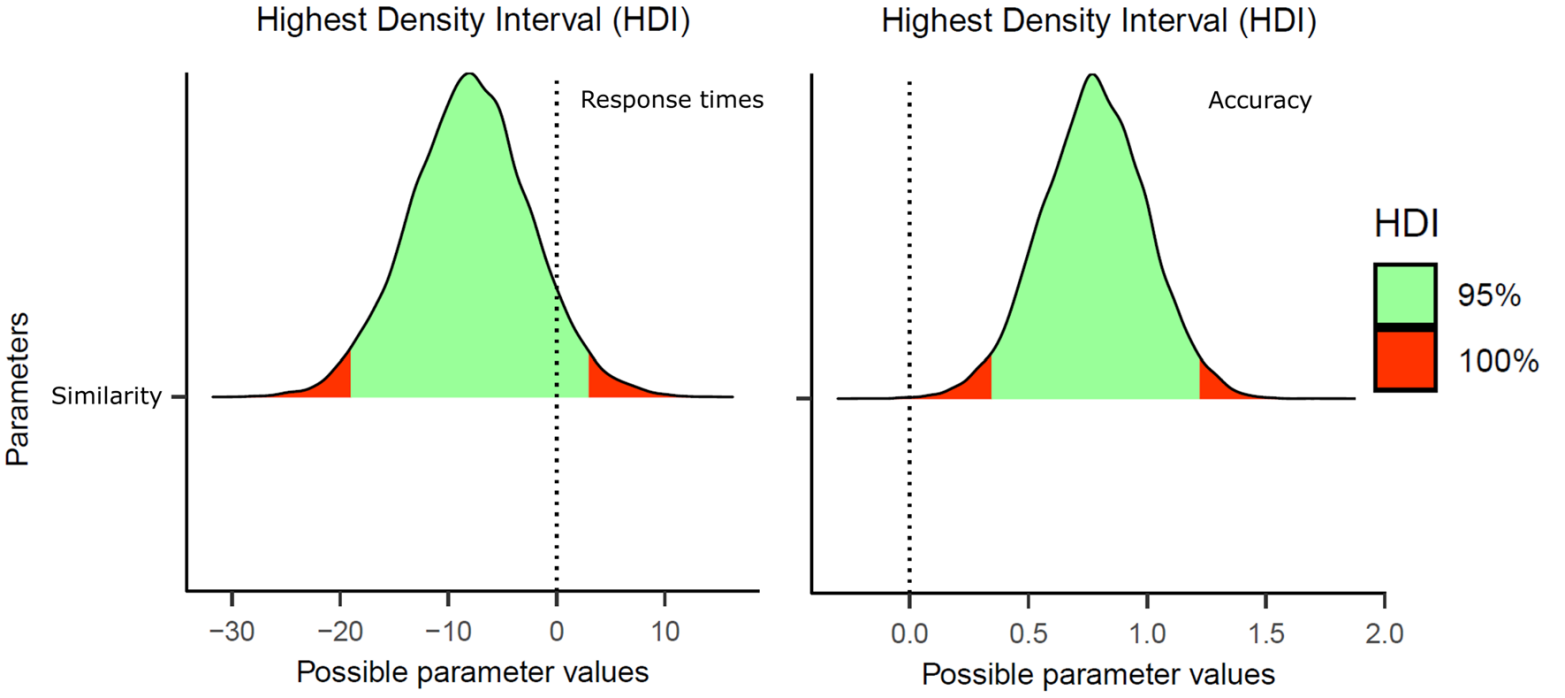
Posterior distributions of the visual letter similarity effect in Experiment 2 (Response Times in the left panel, Accuracy on the right panel). The green areas correspond to the 95% credible intervals

As in Experiment 1, incorrect responses, and response times shorter than 250 ms (0 trials) were removed from the latency analyses. The mean RTs and accuracy in each condition are presented in Table 2. The statistical analyses were the same as in Experiment 1–see Fig. 3 for the posterior distributions of the effects.

**Table 2:** Mean correct response times (in ms) and error rates (in percentage) for the city names and the misspelled city names in Experiment 2 (200-ms exposure duration)

City name	Visually similar misspelled city name	Visually dissimilar misspelled city name	Visual similarity effect
738 (12.0)	784 (18.4)	771 (8.7)	13 (9.7)

#### Response times

The mean RTs for the misspelled city names were, on average, 13 ms slower when the mismatching letter was visually similar to the original letter than when it looked dissimilar—note that the 95% credible interval crossed zero, *b* = − 7.46, *SE* = 5.54, 95% CrI [− 18.46, 3.50].

#### Accuracy

We found evidence of lower accuracy (around 9.7%) for the visually similar misspelled city names than for the visually dissimilar misspelled city names, *b* = 0.75, *SE* = 0.22, 95% CrI [0.32, 1.19].

In the present experiment, briefly presented misspelled city names exhibited a sizeable visual letter similarity effect: participants made more errors with misspelled city names like Barcetona than to Barcesona (see the right panel of Fig. 2). We found an effect in the latency data in the same direction, but it was statistically weaker.

The visual letter similarity effect obtained with misspelled city names in the present experiment poses problems to accounts that assume that abstract letter codes solely drive lexical access (e.g., Dehaene et al., 2005, Local Combination Detector model). Critically, one question raised in this experiment is whether or not the effect of visual letter similarity with misspelled city names under limited viewing time reflects a general process that applies to all misspelled words. To address this issue, we conducted Experiment 3. We selected a set of misspelled common words from the Gutierrez-Sigut et al. (2022) study (e.g., votumen vs. vosumen; base word: volumen [volume]). Gutierrez-Sigut et al. (2022) found that these misspelled words did not show any effects of visual similarity with the standard setup in neurotypical readers in behavioral or electrophysiological measures. Notably, for deaf readers, Gutierrez-Sigut et al. (2022) also reported that misspelled common words like vosumen elicited more negativity in the N400 time window than votumen. Thus, this set of misspelled words may produce visual letter similarity effects, at least for specific populations. (We discuss why some populations may be more sensitive to visual letter similarity effects in the General Discussion.)

There are two possible outcomes in Experiment 3. If the visual similarity effect with misspelled city names found in Experiment 2 reflects a general mechanism for "word" responses based on a partial mismatch between the visual input and the stored representations, we would expect a similar pattern with misspelled common words (i.e., more errors for votumen than for vosumen). Alternatively, if the recognition of common words is primarily based on the rapid activation of abstract letter representations —which would likely occur in less than 200 ms—we would expect similar response times and error rates for visually similar and visually dissimilar misspelled common words (e.g., votumen ≈ vosumen).

## Experiment 3 (misspelled common words, brief presentation)

### Method

#### Participants

We recruited a new sample of 51 participants from the same population as in Experiments 1–2 (mean age: 32.6 years, SD = 11.5, 29 self-identified as women). This sample size guaranteed above 2000 observations per condition, in line with the previous experiments.

#### Materials

We employed the set of words and pseudowords of Gutierrez-Sigut et al. (2022). This set of stimuli contained 120 Spanish words between 5 and 8 letters long (mean frequency per million = 60.58 [range 0.63–888] and mean OLD20 = 1.80 [range 1.05–2.90] in the Spanish EsPal database, Duchon et al., 2013). There were three experimental lists. Each list was composed of 40 intact words (e.g., volumen [volume]), 40 visually similar pseudowords created by changing one middle consonant letter from a base word by a visually similar letter (e.g., votumen; the letter l from volumen was replaced with the letter t; M = 3.28 in the Simpson et al.,’s, 2013, visual similarity matrix), 40 visually dissimilar pseudowords created by replacing the same internal letter as above with a visually dissimilar consonant letter (e.g., vosumen; where the letter l was replaced with the visually dissimilar letter s; M = 1.52 in the Simpson et al., 2013, visual similarity matrix). None of the pseudowords had any word neighbors other than the base word, and the mean log bigram frequencies for the visually similar and visually dissimilar pseudowords were 2.30 and 2.35, respectively. The stimuli were counterbalanced across three lists—the base words were presented intact in the Gutierrez-Sigut et al. (2022) experiment to compare the ERP waves in all three conditions. As in the Gutierrez-Sigut et al. study, each list included 40 filler words to keep a 50% word/pseudoword ratio. We also created ten words and ten pseudowords for the practice phase.

#### Procedure

The procedure was the same as in Experiment 2, except that there was no mention to city names in the instructions.

### Results and discussion

Error responses and RTs shorter than 250 ms (1 trial) were removed from the latency analyses. The mean RTs and accuracy in each condition are presented in Table 3. The analyses were the same as in Experiments 1–2.

**Table 3:** Mean correct response times (in ms) and error rates (in percentage) for the common words and the misspelled common words in Experiment 3 (200-ms exposure duration)

Word	Visually similar misspelled word	Visually dissimilar misspelled word	Visual similarity effect
637 (4.7)	691 (5.0)	699 (5.7)	8 (− 0.7)

**Fig. 4:**
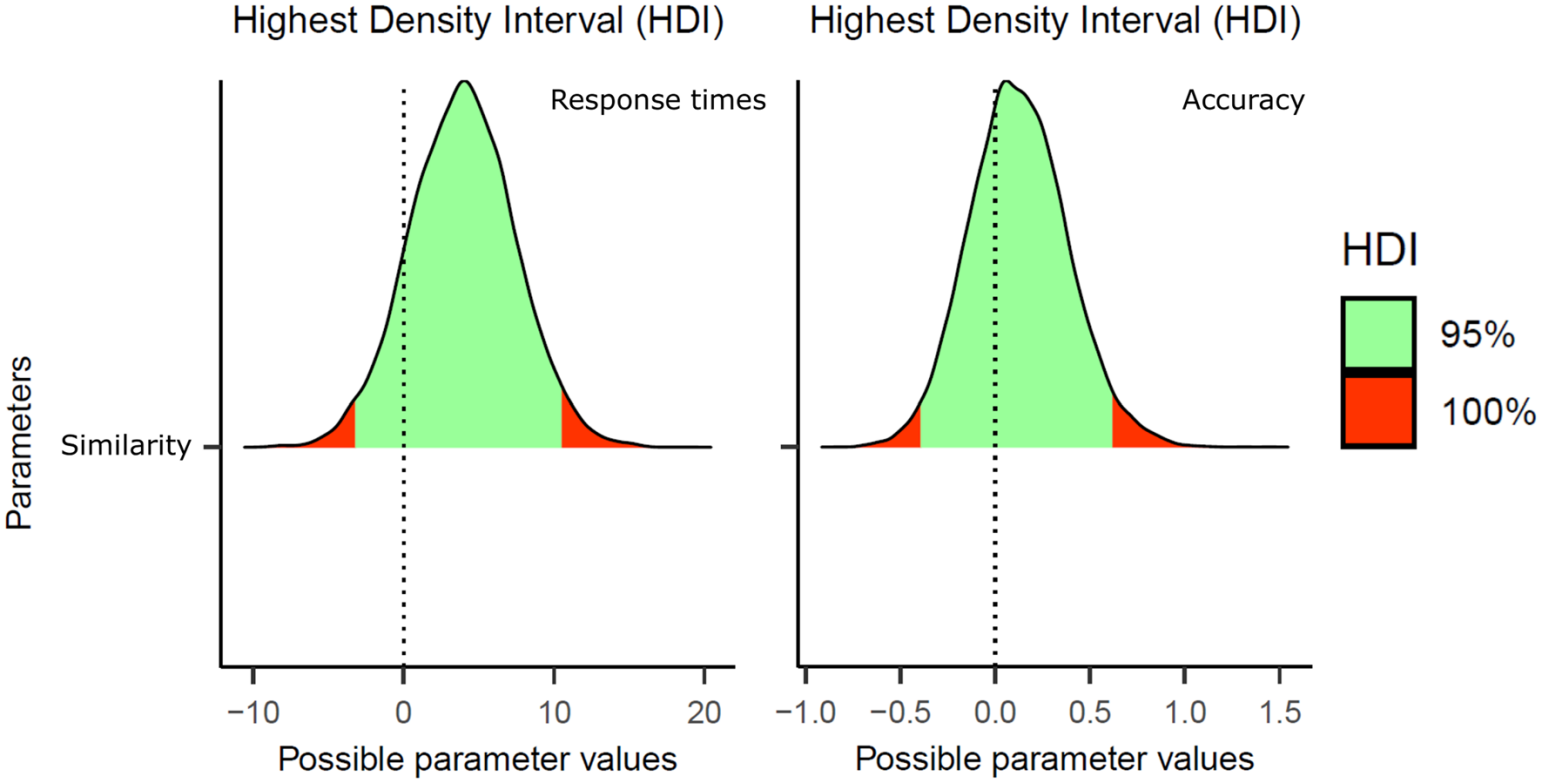
Posterior distributions of the visual letter similarity effect in Experiment 3 (Response Times in the left panel, Accuracy on the right panel). The green areas correspond to the 95% credible intervals

Neither the response times nor the accuracy analyses showed any signs of an effect of visual similarity with misspelled common words with 200-ms exposure durations (RTs: *b* = 3.19, *SE* = 3.49, 95% CrI [− 3.00, 10.78]; accuracy: *b* = 0.12, *SE* = 0.26, 95% CrI [− 0.35, 0.66]) (see Fig. 4 for the posterior distributions; see also the middle panel of Fig. 2).

The present experiment replicated the null effect of visual letter similarity for misspelled common words with skilled adult readers reported by Gutierrez-Sigut et al. (2022) at a shorter exposure duration. Thus, unlike misspelled city names, limiting viewing duration to 200 ms does not elicit a visual letter similarity effect for misspelled common words.

The stimuli used here were taken from a previous study (Gutierrez-Sigut et al. 2022) in which visually similar pseudowords also maintained the overall shape of the original word (e.g., ascending letter → ascending letter, as in votumen [base word: volumen]). The differences in visual letter similarity, as evidenced by Simpson et al.'s (2013) visual letter similarity matrix were large (M = 3.28 vs. 1.52, for visually similar and visually dissimilar pseudowords, respectively; *t*(119) = 13.0, *p* < 0.001). However, the difference between the visually similar and dissimilar conditions in Experiments 1 and 2 was more pronounced (average similarity scores were 4.13 and 1.31 respectively). 

In Experiment 4, we designed a new set of materials involving misspelled common words for which visually similar and visually dissimilar pseudowords more comparable to those of Experiments 1 and 2. That is, in Experiment 4 visually similar pseudowords (e.g., circuilo derived from circuito [circuit]) consistently had high values in visual letter similarity (M = 4.17). In contrast, visually dissimilar pseudowords had low values in visual letter similarity (M = 1.18). In addition, we reduced the stimulus exposure duration from 200 to 150 ms in order to maximize the likelihood of detecting an effect of visual letter similarity for misspelled words, if present.

## Experiment 4 (misspelled common words, brief presentation of 150 ms)

### Method

#### Participants

We recruited 42 participants from the same population as in the previous experiments (mean age: 28.7 years, SD = 8.3, 25 self-identified as women, 16 as men, and 1 as other). As in Experiments 1–3, this sample size guaranteed more than 2,000 observations per condition.

#### Materials

We selected 106 Spanish words between 5 and 8 letters (M = 6.93) to act as base words for the visually similar and visually dissimilar pseudowords. The average frequency per million in the subtitle database in Spanish (Duchon et al., 2013) was 81.1 (range 3.77–470.11) and the mean OLD20 was 1.77 (range 1.00–2.65). For each base word (e.g., circuito [circuit]), we created two pseudowords by replacing an internal consonant letter: (1) with a visually similar letter (visually similar pseudoword; e.g., circuilo; replacing “t” with “l”); (2) with a visually dissimilar letter (visually dissimilar pseudoword; e.g., circuiso; replacing “t” with “s”). The mean similarity of the original letter of the base letter and its visually similar and visually dissimilar counterparts was 4.17 (range 2.53–5.60) and 1.18 (range 1.07–1.40), respectively, in the Simpson et al. (2013) visual letter similarity matrix. None of the pseudowords had any word neighbors other than the base word, and the two sets of pseudowords were matched in mean log bigram frequency (M = 2.26 vs. 2.27 for the visually similar and the visually dissimilar pseudowords; *p* = 0.77). 

In addition, we selected a set of 106 Spanish words between 5 and 8 letters (M = 6.76) to act as word trials in the experiment. The mean word frequency per million was 44.73 (range 1.00–620.78) and the mean OLD20 was 1.82 (range 1.20–3.30). We created two lists, following a Latin-square design, to counterbalance the pseudowords across the two lists (e.g., circuilo would be in List 1 and circuiso would be in List 2). Each list was composed of 53 visually similar pseudowords, 53 visually dissimilar pseudowords, and 106 words.

#### Procedure

The procedure was the same as in Experiments 2–3, except that the script was written in PsyToolkit (Stoet, 2010, 2017), the stimulus duration was slightly shorter (150 ms instead of 200 ms), and the mask was presented for 500 ms followed by a blank screen.

### Results and discussion

We removed very short responses (6 trials, less than 0.001%) and incorrect responses from the latency analyses. Table 3 presents the mean RTs and accuracy in each condition. The analyses were parallel to Experiments 1–3.

**Fig. 5:**
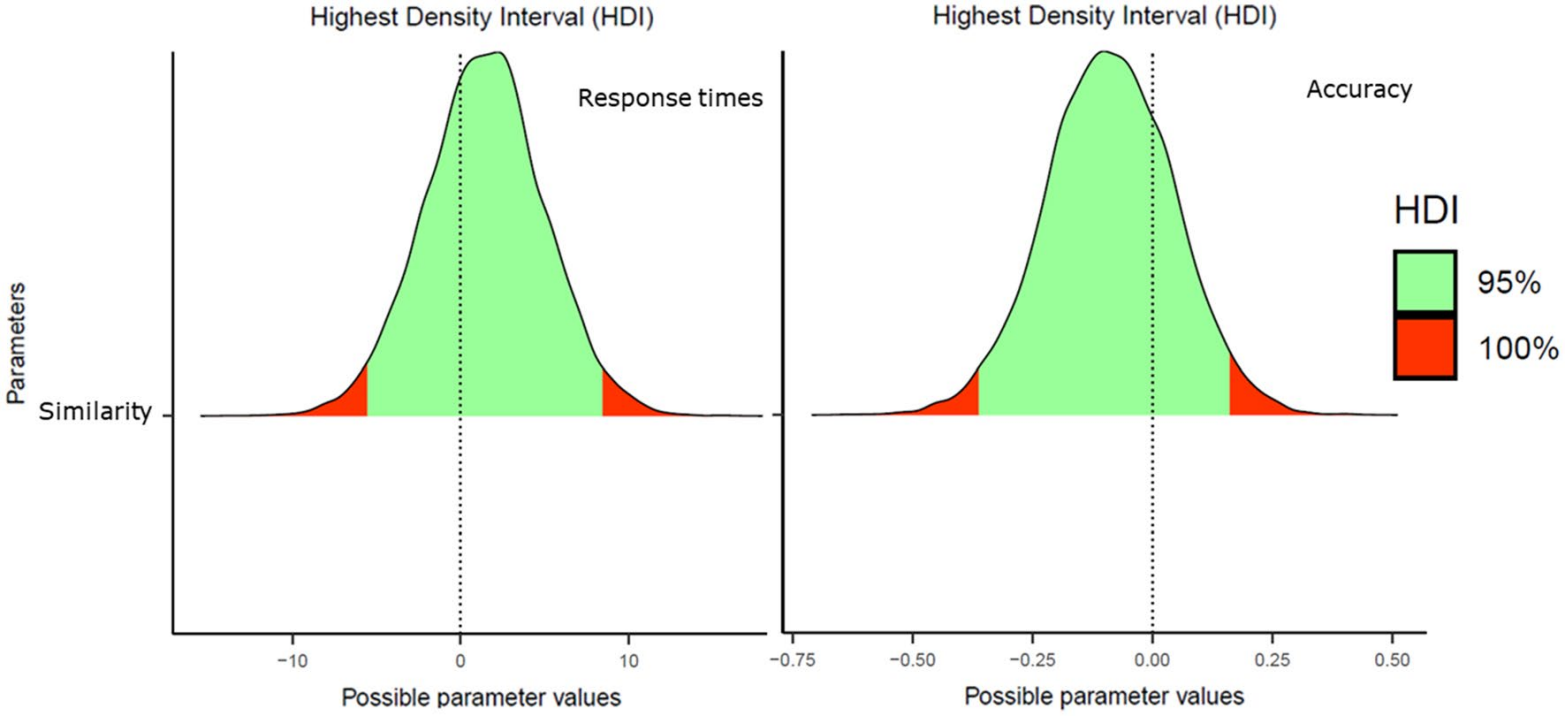
Posterior distributions of the visual letter similarity effect in Experiment 4 (Response Times in the left panel, Accuracy on the right panel). The green areas correspond to the 95% credible intervals

As in Experiment 3, the mean RTs and accuracy rates were comparable for visually similar and visually dissimilar pseudowords (RTs: *b* = 1.41, *SE* = 3.55, 95% CrI [− 5.61, 8.40]; accuracy: *b* = − 0.09, *SE* = 0.13, 95% CrI [− 0.35, 0.17]) (see Fig. 5 for the posterior distributions) (Table 4).

**Table 4:** Mean correct response times (in ms) and error rates (in percentage) for the common words and the misspelled common words in Experiment 4 (150-ms exposure duration)

Word	Visually similar misspelled word	Visually dissimilar misspelled word	Visual similarity effect
609 (8.4)	662 (13.2)	676 (11.9)	− 14 (− 1.3)

The present experiment revealed that, when the stimuli are presented briefly (150 ms), there was no indication of longer response times or increased error rates for visually similar pseudowords relative to visually dissimilar pseudowords in lexical decision (see the right-most plots of Fig. 2). Thus, neither of the experiments with briefly presented misspelled common words revealed any signs of a visual similarity effect, thus extending the findings with the usual setup (e.g., Gutierrez-Sigut et al., 2022; Perea & Panadero, 2014; Perea et al., 2022).

## General discussion

One central question in visual-word recognition and reading is whether lexical access in skilled readers is based solely on abstract codes—as typically assumed—or whether visual codes may also play a role. The robustness of the visual similarity effects for misspellings of logotypes and brand names (e.g., the visually similar misspelling brand name amazom [from amazon] is often identified as a legit brand name; see Pathak et al., 2019; Perea et al., 2022) poses problems for those models of visual-word recognition proposing purely abstract codes. To explain this dissociation, one might argue that the processing of logotypes and the context in which they occur are very different from other categories of words because of the consistency in their format. In the present experiments, we investigated whether another type of stimuli—city names—is sensitive to visual similarity effects by comparing visually similar pseudowords like Barcetona vs. visually similar pseudowords like Barcesona in lexical decision. The logic was that, although to a lesser degree than brand names, city names are usually presented in a more homogeneous format than common words (e.g., initial capitalization, often in print). Using the standard setup (i.e., stimulus presentation until the participant's response), Experiment 1 found no evidence of visual similarity with misspelled city names (e.g., Barcetona vs. Barcesona). Crucially, when post-access verification mechanisms were restricted via a limited viewing time (i.e., stimulus presentation of 200 ms), we observed more errors for visually similar pseudowords (e.g., Barcetona) than for visually dissimilar pseudowords (e.g., Barcesona). Importantly, we also conducted two other experiments (Experiments 3 and 4) to test whether a limited viewing presentation would elicit a parallel effect with misspelled common words. Using different sets of items and varying stimulus presentation duration (200 ms in Experiment 3 and 150 ms in Experiment 4), we found no evidence of a visual letter similarity effect for misspelled common words.

The findings of Experiment 2 with misspelled city names challenge those models of visual-word recognition that posit that visual information is entirely abstracted at the initial stages of letter encoding (e.g., Dehaene et al., 2005, Local Combination Detector model). Instead, the word recognition system may maintain at least some sensitivity to visual forms throughout processing (see Carreiras et al., 2013, for electrophysiological evidence with isolated letters). This sensitivity to visual information would be crucial for words we often encounter in a similar visual form, such as brand names and logotypes (Pathak et al., 2019; Perea et al., 2022). Notably, we have demonstrated that these visual codes also play a role in another category of words—city names—that is usually presented with a homogeneous format. These findings support the idea that the processing of written words may lead to multiple access codes, some of which may retain some perceptual characteristics (e.g., see Davis, 1999; Hannagan et al., 2021; Henderson, 1987; Peressotti et al., 2003; Wimmer et al., 2016). Thus, visual codes may play a more prominent role in visual word recognition for certain types of stimuli like brand names and—to a lesser degree—city names, and for certain populations. For example, dyslexic readers and deaf readers show visual letter similarity effects with misspelled common words (see Gutierrez-Sigut et al., 2022; Perea & Panadero, 2014; see also Lavidor, 2011), while these same items do not produce any effect in typical readers. This dissociation across neurotypical versus dyslexic or deaf populations has often been attributed to differences in the balance between visual and abstract codes (see Emmorey et al., 2021; Lavidor, 2011).

Thus, an explanation of the present findings—together with the results with brand names—is that the brain stores sensory information from previous encounters with the words (see Goldinger, 1998, for an episodic account of lexical access). The blended mental representations of common words would result from a wide range of variability in the sensory format, so that their representations would not be typically affected by visual letter similarity beyond the initial stages of letter encoding. Conversely, the representations of highly homogeneous stimuli like brand names would be particularly sensitive to deviant visual elements (e.g., anazon would activate amazon more than atazon). In addition, the representation of stimuli that keep some homogeneity in the visual format, like city names can be susceptible (to a lesser degree) to visual elements (e.g., Barcetona would activate Barcelona more than Barcesona, at least with relatively brief exposure durations). Interestingly, this interpretation can easily explain why misspellings in common words in braille produce a tactile letter similarity effect (Baciero et al., 2022): braille letters have a characteristic homogeneous format (e.g., see UK Association for Accessible Formats, 2017), thus making them more sensitive to perceptual effects.

There is another explanation. Recent models of visual-word recognition have been moving towards scenarios that do not use explicit abstract letter representations. Agrawal et al. (2020) implemented a neurally plausible, purely visual model based on the compositional code of individual letters that respond to letter shapes. In this model, “lexical decisions for nonwords are driven by the dissimilarity between the viewed string and the nearest word” (p. 13). Therefore, the visually similar pseudoword forcet would be more confusable with word forget than the word forxet (see Fig. 2 in Agrawal et al., 2020). Thus, this model can easily explain the presence of visual letter similarity effects for misspellings in lexical decision. The problem is that, for common words, these visual similarity effects only appear with heavily masked stimuli (e.g., masked primes: Marcet & Perea, 2017, 2018; Reicher-Wheeler task: Lally & Rastle, 2022). Furthermore, visual letter similarity effects are substantially greater for misspelled brand names than for misspelled city names for unprimed lexical decision. Future implementations of the models based on compositional codes need to consider the role of the variability across the visual input in their learning regime. We must keep in mind that Agrawal et al.’s model was trained with stimuli with the same font, thus creating a scenario similar to that of brand names and, thereby, especially susceptible to the influence of visual factors. More recently, Hannagan et al. (2021) implemented a deep convolutional neural network that was originally trained to perform object recognition and was later trained with thousands of images of words varying in case, font, and size. This model predicted a number of phenomena previously attributed to the emergence of abstract letter representations (see also Yin et al., 2022, for another convolutional network model that simulates masked form priming; see also Bowers et al., 2022, for discussion of these networks). However, unlike Agrawal et al.’s (2020) proposal, Hannagan et al. (2021) did not test visual similarity effects in lexical decision. While beyond the scope of the present paper, further simulations with these models are necessary to examine their plasticity to the various types of items (e.g., brand names, city names, common words) or groups of participants (e.g., readers with dyslexia).

In sum, the present experiments revealed that visually similar misspellings like Barcetona were more difficult to reject as words than Barcesona under relatively brief exposure durations (200 ms). This pattern did not occur with misspelled common words in two additional experiments using different sets of items. This dissociation suggests that, at least for some types of words, visual codes are used during word processing flow, challenging biologically-inspired models of visual word recognition that rely solely on the activation of abstract letter codes (e.g., Dehaene et al.,’s 2005, Local Combination Detector model). Future implementations of models of visual-word recognition should consider that words that are often presented in a homogeneous format (e.g., brand names and, to a smaller degree, city names) can be more sensitive to visual codes than common words.

## Data Availability

The stimuli, data, scripts, and outputs are available at https://osf.io/drsvu/?view_only=c3bd69188768472f86fc6b721997950f. Experiments 1 and 2 were pre-registered at https://osf.io/js5r7/?view_only=68bea8601f394402b11d08b1b42ab919 and https://osf.io/xbczm/?view_only=3125ab7097ee48aab4a5115fa6971822

## Footnotes

1 We acknowledge that another option would have been to use proper names (e.g., see Peresotti et al., 2003; Sulpizio & Job, 2018). We chose city names because, in general, they tend to have a more consistent visual format than proper names (e.g., children often use handwriting to write and read theirs and their peer’s names, yielding more variability in the sensory input).
